# A Spatially Variable Time Series of Sea Level Change Due to Artificial Water Impoundment

**DOI:** 10.1029/2020EF001497

**Published:** 2020-07-25

**Authors:** William B. Hawley, Carling C. Hay, Jerry X. Mitrovica, Robert E. Kopp

**Affiliations:** ^1^ Department of Earth and Planetary Science University of California, Berkeley Berkeley CA USA; ^2^ Berkeley Seismological Laboratory University of California, Berkeley Berkeley CA USA; ^3^ Now at Lamont‐Doherty Earth Observatory of Columbia University Palisades NY USA; ^4^ Department of Earth and Environmental Sciences Boston College Chestnut Hill MA USA; ^5^ Department of Earth and Planetary Sciences Harvard University Cambridge MA USA; ^6^ Department of Earth and Planetary Sciences and Rutgers Institute of Earth, Ocean, and Atmospheric Sciences Rutgers University New Brunswick NJ USA

**Keywords:** sea level, reservoirs

## Abstract

The artificial impoundment of water behind dams causes global mean sea level (GMSL) to fall as reservoirs fill but also generates a local rise in sea level due to the increased mass in the reservoir and the crustal deformation this mass induces. To estimate spatiotemporal fluctuations in sea level due to water impoundment, we use a historical data set that includes 6,329 reservoirs completed between 1900 and 2011, as well as projections of 3,565 reservoirs that are expected to be completed by 2040. The GMSL change associated with the historical data (−0.2 mm yr^−1^ from 1900–2011) is consistent with previous studies, but the temporal and spatial resolution allows for local studies that were not previously possible, revealing that some locations experience a sea level rise of as much as 40 mm over less than a decade. Future construction of reservoirs through ~2040 is projected to cause a GMSL fall whose rate is comparable to that of the last century (−0.3 mm yr^−1^) but with a geographic distribution that will be distinct from the last century, including a rise in sea level in more coastal areas. The analysis of expected construction shows that significant impoundment near coastal communities in the coming decades could enhance the flooding risk already heightened by global sea level rise.

## Key Points

- We present a spatially resolved time series of sea level due to reservoir construction from 1900–2040
- Some locations in the past century have experienced a reservoir‐induced rise in sea level that exceeds 40 mm over less than a decade
- Future dam construction will cause patterns in sea level change that are different from the past and larger than current projections

## 1 Introduction

Global and regional changes in sea level are driven by a wide range of processes, including the redistribution of mass from melting glaciers and ice sheets, the continued adjustment of the solid Earth to ice mass changes during the last glacial period, steric expansion of ocean water, ocean circulation changes, and both natural and artificial changes in terrestrial water storage (Cazenave & WCRP Global Sea Level Budget Group, 2018; Kopp et al., 2015). Proper characterization of each of these different components of the sea level budget is necessary for accurate projections of future changes in sea level. In this context, the role of terrestrial water storage remains underexplored and serves as the focus of the present study. We provide the most complete picture to date of the impact that artificially impounded water has had on sea level over the last century, resolved both spatially and temporally, and an estimate of future sea level changes due to projected reservoir construction.

Chao et al. (2008) use the International Commission on Large Dams World Register of Dams (WRD) (www.icold‐cigb.org) database to construct an estimate of the global mean sea level (GMSL) change due to the construction of 29,484 reservoirs worldwide. They estimate the total volume of impounded water to be 10,800 km^3^, corresponding to a GMSL fall of ~0.55 mm yr^−1^ in the half century prior to their publication. Sea level changes associated with the impoundment of water on land will be geographically variable (Fiedler & Conrad, 2010), and each reservoir will have a unique sea level “fingerprint,” or gravitational, rotational, and deformational (GRD) response to mass redistribution (Gregory et al., 2019). The redistribution of water from the ocean to the reservoir will (1) increase the gravitational attraction of the reservoir on the surrounding water and thus raise the local sea surface height, (2) induce a change in Earth's moment of inertia, and (3) drive local crustal subsidence. Indeed, relative sea level (RSL) will rise within ~2,000 km of a reservoir being filled, despite a drop in GMSL, and it will fall by increasing amounts at larger distances from the source of impoundment. The local signal, which can have a peak value an order of magnitude larger than the GMSL change associated with the impoundment, is primarily a result of processes (1) and (3). Calculating the global sea level pattern associated with water impoundment requires knowledge of both the size and location of a reservoir.

The WRD database adopted by Chao et al. (2008) includes the largest global tabulation of reservoirs and the most complete estimate of the total volume stored in those reservoirs. However, it does not provide locations for these reservoirs and thus cannot be used to generate maps of the associated sea level change. To compute such a map, Fiedler and Conrad (2010) adopt a data set (Vörösmarty et al., 1997) that includes the locations of 674 reservoirs currently built and scale their result upwards to match the GMSL cited by Chao et al. (2008). We extend their analysis in three ways. First, we make use of a much larger database of reservoirs. Second, we explore both the spatial and temporal patterns of RSL change associated with water impoundment. Finally, we project the signals into the future using a database of planned dam construction (Zarfl et al., 2015).

## 2 Reservoir Databases

**Figure 1:**
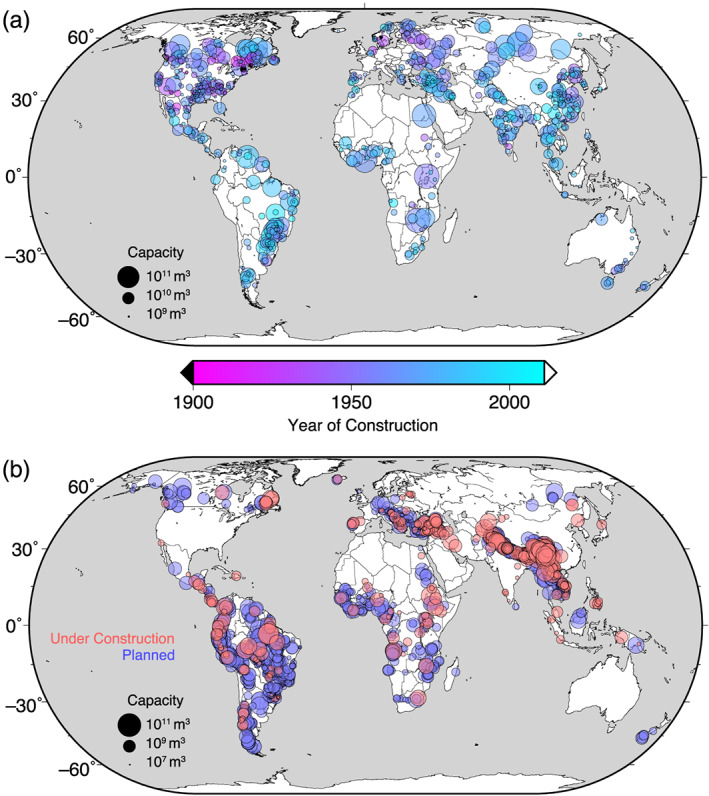
Map of reservoirs and their capacities. Global maps of reservoir locations, with the size of symbols corresponding to (log) capacity, and color corresponding to year of completion. Only larger reservoirs are plotted. Panel (a) shows the GRanD database (Lehner et al., 2011a, 2011b); panel (b) shows the database of Zarfl et al. (2015).

**Figure 2:**
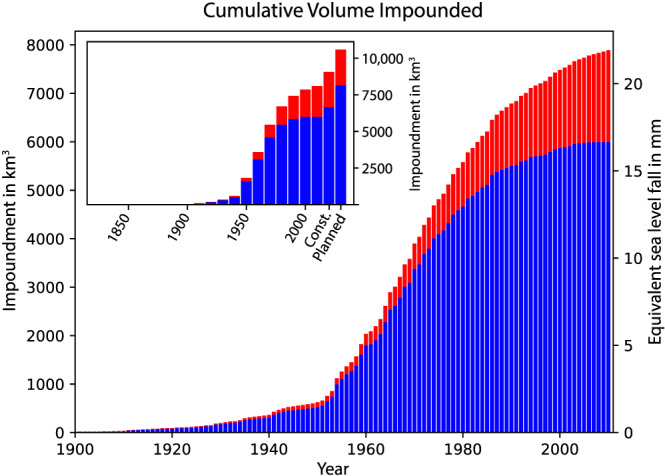
Histogram of total volume of water impoundment. Main figure: Total integrated volume of water impounded as a function of time based on the GRanD database without (blue) and with (red) seepage considered. Right axis shows the equivalent GMSL fall associated with the impoundment. Inset: Extension of both curves in the main figure to include projected water impoundments for reservoirs under construction (“Const.”) and Planned. Seepage for the column labeled “Const.” is estimated only from the GRanD database to the year 2025. Seepage for the column labeled “Planned” is estimated from the GRanD database to the year 2035, plus 10 years of seepage from those reservoirs in the “under construction” category.

In the present study, we use two different databases of reservoir construction. For the time period 1900–2011, we use the Global Reservoir and Dam (GRanD) database (Lehner et al., 2011a, 2011b), which aims to geospatially reference all reservoirs with a capacity of more than 0.1 km^3^. It contains 6,329 reservoirs that were completed after 1900 and reports their location, year of construction, and capacity (Figure 1a). The total volume of these reservoirs is 5,979 km^3^, which is less than 8,300 km^3^, as reported in the WRD (Chao et al., 2008). Time series of integrated water volume impoundment (and equivalent GMSL fall) for the GRanD database is shown in Figure 2 (blue histogram). Although the GRanD database contains only ~72% of the volume reported in the WRD, we do not scale our total impounded water volume to match the WRD values because the primary purpose of this study is to estimate the spatial variability of sea level, and water impoundment not included in the GRanD database has an unknown geographical distribution.

In the GRanD database, standard filling rates suggest that we can assume that the smaller reservoirs filled over the course of a year. However, the same is not true for the largest reservoirs in the database. In the case of the largest nine reservoirs, we use various records of the filling duration or average discharge rate (e.g., Loo, 2007) and assume a steady filling rate. For these nine largest reservoirs, the average volume added per year is approximately 23 km^3^. The 49 largest reservoirs in GRanD database have a capacity larger than 23 km^3^, and for all these reservoirs, we adopt a filling rate of 23 km^3^ yr^−1^. Each reservoir smaller than this is assumed to have filled the year the dam was completed. These filling rates are accounted for in the time series of Figure 2.

For the future projections, we use a database compiled by Zarfl et al. (2015), which includes dams that will be built for hydropower and whose power generation capacity exceeds 1 MW (Figure 1b). The data set contains no direct information on the expected impounded volume of each reservoir; however, it lists the planned hydroelectric capacity. We use this value to estimate the impounded volume, following Grill et al. (2015):

(1) $$V=\left(3.19\times 10^{6}m^{3}\;{\mathrm{MW}}^{-1}\right)P,$$

where *V* is the reservoir volume and *P* is the power generation capacity. Furthermore, because these dams were not complete at the time of the publication of Zarfl et al. (2015), many do not have an estimated year of completion. Instead, 3,565 reservoirs are listed as being in one of two broad categories, either in the construction phase (*n* = 501, or 15% of the total) or the planning phase (*n* = 2,894, or 85%). Using these categories and the equation above, we estimate that the reservoirs under construction will impound 663 km^3^ and that those being planned will contribute an additional 1,500 km^3^. Adding these values to results for the GRanD database yields the time series of integrated water volume impoundment shown in the inset to Figure 2, assuming, following Zarfl et al. (2015), that all dams will be completed by the year 2040 (blue histogram).

## 3 Results

### 3.1 Temporal Variation in GMSL

Removing 5,979 km^3^ of water from 1900 to 2011 according to the GRanD database corresponds to a GMSL fall of 16.6 mm, or an average 0.15 mm yr^−1^. As noted above, these values are 72% of the 23 mm and 0.21 mm yr^−1^ reported in Chao et al. (2008). Impounding the 663 km^3^ (under construction) and 1,500 km^3^ (planned) from the Zarfl et al. (2015) database will cause a further globally averaged drop in sea level of 1.8 and 4.2 mm, respectively, for a total of 6 mm. Thus, over the period 2020–2040, the mean GMSL rate associated with water entering reservoirs will likely be −0.3 mm yr^−1^, which is larger than the average rate that occurred over the past century.

The above numbers will increase when accounting for natural seepage of water into the land surrounding the reservoir. After a reservoir is built, water will slowly seep into the neighboring land. As this occurs, the flow of water into the reservoir continues to recharge it, but the water that seeps from the reservoir remains relatively localized and does not generally reach the ocean, adding to the total impounded water. While the rate of seepage depends on numerous local factors (e.g., Harr, 1962), for simplicity, we follow Chao et al. (2008) and use a single equation to estimate the seepage rate at each reservoir:

(2) $$V\left(t\right)=0.05\;V_{0}\;t^{1/2},$$

where *V*_0_ is the reported volume of the reservoir, *t* is the time in years since the water was impounded, and *V*(*t*) is the total amount of water that seeps by time *t*. According to this equation, seepage contributed an estimated 1,878 km^3^ of water by 2011, for a total impounded volume of 7,857 km^3^. This corresponds to a total GMSL drop of 21.8 mm from 1900–2011 (Figure 2, red histogram in main frame and inset). By 2040, we estimate that this value will increase to 30.1 mm, assuming for the seepage term that every “under construction” reservoir is completed by 2025 and every “planned” reservoir is completed by 2035. (We make the assumption that, for example, every “planned” reservoir is constructed in the decade 2030–2039; assigning every “planned” reservoir to the year 2039 will result in a seepage rate that is too low. We choose a date in the middle of the decade to give the best sense of the GRD fingerprint including seepage.)

Analysis of the GRanD time series indicates that the GMSL time series is characterized by markedly different rates in three distinct time windows: from 1900 to 1949, the volume of impounded water rose gradually; from 1950 to 1979, the rate of water impoundment increased dramatically; and from 1980 to 2011, new construction slowed but seepage became increasingly significant. Predictions from the Zarfl et al. (2015) database show a further dramatic increase in water impoundment in the next two decades. Many of these reservoirs will be near the coast (Figure 1b), so a complete characterization of the effect this impounded water will have on sea level will help refine coastal hazard assessment.

### 3.2 Spatial Variation in Sea Level

For each of the 9,724 records in our combined database, we generate a gravitationally consistent prediction of the GRD fingerprint by solving a version of the so‐called sea level equation (Farrell & Clark, 1976) that includes the feedback into sea level of impoundment‐induced perturbations in the Earth's rotation vector and shoreline migration (Mitrovica & Milne, 2003), although the latter is negligible in the calculations performed here. The results are generated using the pseudo‐spectral algorithm of Kendall et al. (2005) applied to a 1‐D elastic Earth model in which an initial guess to the fingerprint is iteratively improved until convergence is reached. Three such iterations are generally sufficient to establish convergence. Mitrovica et al. (2011) have shown that our neglect of lateral variations in mantle density and elastic constants, which is motivated by the high computational requirements of 3‐D simulations, introduces a small, 1% error in the fingerprints. We adopt the depth‐varying elastic and density structure reported by the seismically inferred Preliminary Reference Earth Model (Dziewonski & Anderson, 1981), and the calculations are based on a spherical harmonic truncation at degree and order 512. Test calculations at tide gauge sites using a spherical harmonic truncation level of 1024 show negligible differences with those reported below.

An example GRD fingerprint is shown for Manicouagan Reservoir (the sixth largest in our data set, with a capacity of 162 km^3^) in Figure S1 in the supporting information. The amplitude of the sea level rise close to the reservoir exceeds the GMSL changes associated with this impoundment by over an order of magnitude, and the zone of predicted sea level rise extends ~2,000 km from the location of the reservoir. In the far‐field of the reservoir, the predicted sea level fall reaches an amplitude ~35% greater than the GMSL change. Moreover, the signal due to rotational effects is evident in the global fingerprint (note in Figure S1 the decreased magnitude south of Australia and the increased magnitudes over eastern Asia and southern South America).

**Figure 3:**
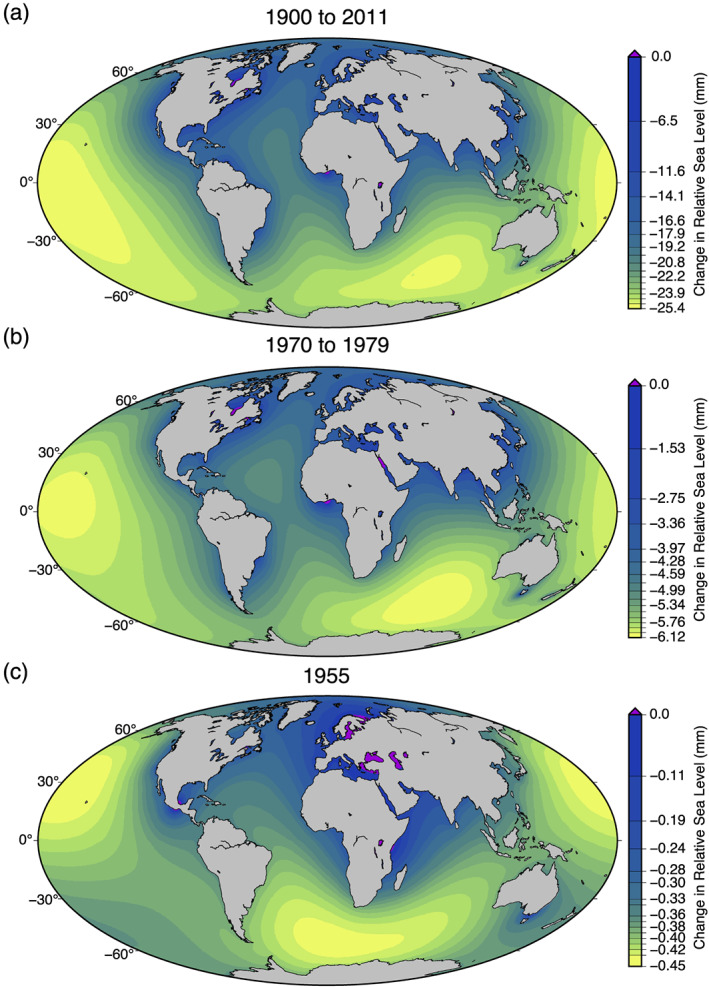
Sea level GRD fingerprints over various time periods showing the relative sea level change as a result of reservoir construction including seepage. In each plot, the color bar is scaled to the maximum sea level fall. (a) Combined fingerprint of all reservoirs in the GRanD database (1900–2011), including seepage. (b) Fingerprint of all water impounded in the decade 1970–1979, a period of prolific dam construction. (c) Fingerprint of all water impounded during the year 1955.

In the absence of significant shoreline migration, the computed GRD fingerprints add linearly. Taking advantage of this linearity, we have constructed a net sea level fingerprint for every calendar year, accounting for the various time series of reservoir impoundment and seepage rates. Fingerprints for a selected set of years are shown in Figure 3. The full suite of annual fingerprints for the period 1900–2011 is available at doi.org/10.5281/zenodo.3751986. A comparison with the fingerprint of Fiedler and Conrad (2010) is shown in Figure S2.

**Figure 4:**
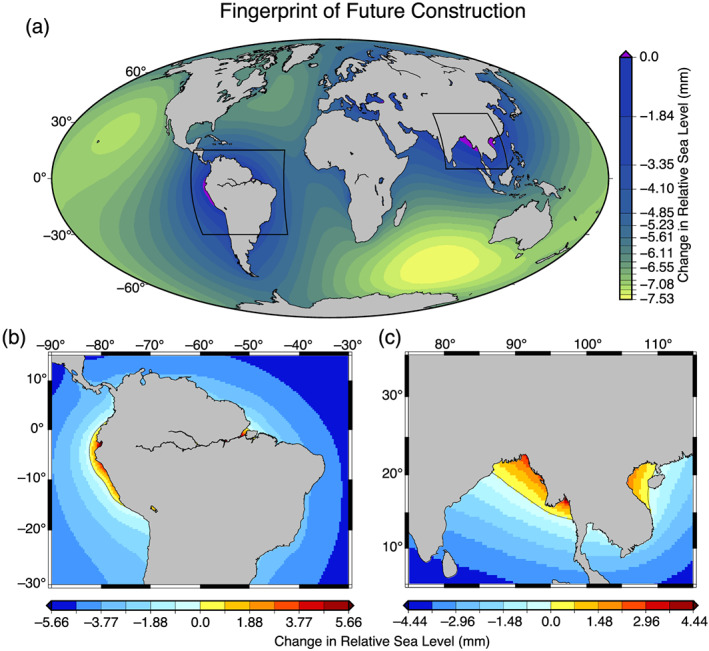
Relative sea level change computed using all reservoirs in the Zarfl et al. (2015) database, assumed to be completed by 2040, with seepage included as described in the text. (a) Global GRD fingerprint signal. Colors as in Figure 3. (b) Detail of frame (a) in South America, showing sea level rise of a few mm along the west coast of Ecuador and Peru and around the mouth of the Amazon River in Brazil. Cool colors show sea level fall; warm colors show sea level rise. (c) Detail of frame (a) in Southeast Asia, indicating sea level rise of a few mm along the low‐lying coasts of Bangladesh and Myanmar and 1–2 mm along the Vietnamese coast along the Gulf of Tonkin.

The GRD fingerprints for the future construction from the database of Zarfl et al. (2015) are shown in Figure 4. The global signal (Figure 4a) shows a markedly different geographic distribution than the GRanD database (Figure 3a), notably in northern South America and Southeast Asia.

## 4 Discussion

### 4.1 Tide Gauge Observations

**Figure 5:**
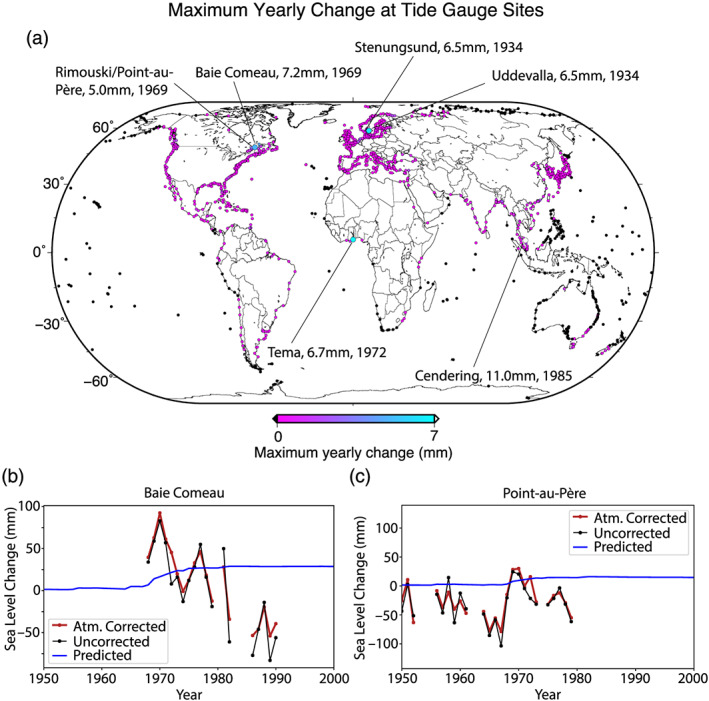
Tide gauge predictions and observations. (a) Maximum yearly change at each PSMSL RLR tide gauge site. Six sites have a predicted a maximum yearly change of 5 mm or more. These six sites are distinguished by a larger symbol; also indicated are the tide gauge name, the maximum predicted yearly increase, and the year in which that maximum occurred. (b) Predicted sea level change due to reservoir construction (blue) versus RLR PSMSL observed (black dots) and atmospheric‐corrected sea level (Piecuch et al., 2019; red points) at the Baie Comeau, Québec, Canada, tide gauge, near the Manicouagan Reservoir. The reservoir was filled from 1970 to 1978. Units on the vertical axis are millimeters, but the absolute value is arbitrary. Panel (c) as in panel (b), but for tide gauge in Point‐au‐Père, Québec, Canada.

Our time series provides a globally resolved estimate of the yearly change in sea level from 1900–2011 due to the construction of reservoirs. Tide gauges record local changes in sea level, which may show the effect of reservoir construction. Because of the variability in sea level recorded in tide gauge data, only the largest, most rapid changes will be visible. These are likely to be not the result of sustained building of reservoirs over time, but rather a tide gauge's proximity to a single large reservoir. To determine whether any of these changes are resolvable in the tide gauge record, we first calculate the maximum yearly rate of change predicted at each tide gauge site in the Permanent Service for Mean Sea Level (PSMSL) revised local reference (RLR) database (http://www.psmsl.org/data/obtaining/; Holgate et al., 2013), shown in Figure 5a. The largest predicted changes in sea level due to water impoundment are in northern Europe, Ghana, Malaysia, and the Saint Lawrence Seaway in Canada, all of which have tide gauges in close proximity (< 200 km) to the construction of a single large reservoir.

We begin by correcting the tide gauge data using two barotropic ocean circulation models (Piecuch et al., 2019). These models predict sea surface height from surface wind stress and air pressure records from two forcing data sets, ERA‐20C (Poli et al., 2013) and NOAA‐20CRv2 (Compo et al., 2011), and extend throughout the 20th century. At each tide gauge location, we subtract the annual average sea surface height derived in these models at each candidate tide gauge location from the raw tide gauge data to remove the atmospheric contribution from the sea level records. We then compare the atmospheric‐corrected sea level time series to our predicted changes due to historical impoundment, focusing on locations where we predict that the largest water impoundment‐induced signals occurred.

The largest predicted rates of change due to impoundment occur at two tide gauges in the St. Lawrence River in Canada, Baie‐Comeau and Point‐au‐Père/Rimouski. The filling of the Manicouagan reservoir from 1968–1974 is predicted to have caused a sea level rise at these sites of almost 40 mm over 6 years (more than 6 mm yr^−1^, which is more than 7 times the GMSL rise over this period, 0.8 mm yr^−1^; Dangendorf et al., 2019) The expected, observed, and corrected tide gauge records are shown in Figures 5b and 5c. There is too much variability in the observations to confidently detect the signal associated with the construction of the Manicouagan reservoir in the tide gauge data. In any case, the St. Lawrence River is a controlled waterway, so the impoundment signal in the record may be muted or entirely absent. Other sites were either not in operation at the right time (e.g., Stenungsund, Sweden; Tema, Ghana; and Cendering, Malaysia). Candidate tide gauge predictions and observations are shown in Figure S3. A more complete characterization of sea level behavior, whether through using a variety of ocean reanalysis models (Chepurin et al., 2014) or more sophisticated modeling techniques (Piecuch et al., 2017), may resolve an impoundment signal in tide gauge data sets.

### 4.2 Variability in Reservoir Storage

Seepage is not the only mechanism that can alter the mass of a reservoir after impoundment. An additional postconstruction signal comes from variations in the amount of water impounded, either seasonally or in long‐term drawdown of the water. We assess both of these for a handful of the largest reservoirs. Records of lake levels can be difficult to obtain in a uniform manner, so we use changes in the surface height as observed by satellite altimetry, provided by the U.S. Department of Agriculture (USDA) Global Reservoir and Lake Elevation Database (https://ipad.fas.usda.gov/cropexplorer/global_reservoir/) to estimate the mass change. The satellites generally observed the largest reservoirs 2 to 3 times a month, beginning in 1992 and continuing through the study period. Because fluctuations in lake level are small over the ~10‐day time scale between satellite observations, the behavior of the reservoir volume can be reasonably well constrained by this method. Dramatic increases of impounded water that happen over a shorter time scale are generally due to large precipitation events, in which case the signal from the reservoir is likely to be masked by a significant increase in groundwater and surface water unrelated to the reservoir itself.

From the surface area and volume provided for each reservoir in the GRanD database, we calculate a mean depth. The altimetry data show changes in altitude, but not absolute altitude, so we cannot use these data to verify when the reservoir is full. Instead, we set the maximum datum from the altimetry as the mean depth of the reservoir, assuming that this represents a full reservoir. We then use every other altimetric data point to estimate the fractional change of the mean depth and approximate this as the fractional change of the volume of the reservoir. We discuss both long‐term drawdown and seasonal changes in water storage.

Lake Powell in the United States is an excellent target for detailed investigation. First, high‐resolution data are available to verify the accuracy of the GRanD data set. Second, satellite altimetry data show that Lake Powell is characterized by a decline in lake level of slightly more than 12 m between 2000 and 2011. Therefore, we can use the data set to investigate the effects of a long‐term reduction in water storage and whether the use of satellite altimetry is appropriate in determining storage changes in large reservoirs.

GRanD lists Lake Powell as the 43rd largest reservoir in the database, with a capacity of 25.07 km^3^ and a surface area of 120.7 km^2^. In contrast, the Bureau of Reclamation (BoR; part of the United States Department of the Interior), which manages Lake Powell and the Glen Canyon Dam that impounds it, lists the capacity as 32.3 km^3^ and the surface area as 688.9 km^2^ (https://www.usbr.gov/uc/rm/crsp/gc/). These two sets of estimates produce very different values for the mean depth of the reservoir, 208 and 47 m, respectively. The 12‐m decline shown in the satellite altimetry data, then, represents a reduction in impounded water of either 6% or 25%. The BoR also provide daily calculations of reservoir storage based on the observed elevation of the water level and bathymetric surveys. For the time period 2000 to 2011, these surveys (https://www.usbr.gov/rsvrWater/HistoricalApp.html) indicate that the elevation in Lake Powell decreased 13 m from 1,122 to 1,109 m, and that storage in Lake Powell decreased from 27.1 to 19.7 km^3^, a decrease of 27%. The satellite altimetry data are thus consistent with the water impoundment reported by the BoR and additionally support the BoR‐reported parameters for reservoir size rather than the values in the GRanD database.

While a decline of 27% in the volume of the reservoir does contribute meaningfully to the GRD fingerprint through time, it is similar in magnitude—but opposite in sign—to the seepage we assume for Lake Powell (Figure S4b). This time series indicates that significant, long‐term drawdown in impoundment can change the magnitude of local sea level change associated with the unloading. However, for reservoirs that do not experience significant drawdown in storage, periods of low water levels will be characterized by a relatively small perturbation in local sea level, and will have a second‐order effect on the global fingerprint of artificially impounded water.

Lake Guri, a reservoir in Venezuela with a volume of 135 km^3^, experiences seasonal variations in surface height that routinely exceed 10 m, representing almost 30% of the reservoir's capacity as calculated from the GRanD‐reported volume and surface area. We compare sea level predictions—those that do and do not include variability in Lake Guri—at the closest grid point to Lake Guri at in Figure S4a. Note that this location is not on the coast; because the coast is at a greater distance, the impoundment signal will be smaller there. The perturbations in sea level in this case are on the order of a few millimeters, with higher sea level occurring when the reservoir is full, as expected. If the seasonality is due to precipitation, that is, a full reservoir occurs during a rainy season, then GRD effects associated with the higher level of impoundment could enhance the hazard of coastal flooding at precisely the time when increasing rain makes flooding more likely.

### 4.3 Future Changes in Sea Level

Our projections of future changes in sea level due to water impoundment are significantly larger than previous estimates. Following Rahmstorf et al. (2012), Kopp et al. (2014) use impoundment data from the WRD (Chao et al., 2008) and population projections from the United Nations Department of Economic and Social Affairs, Population Division (2013) to derive a relationship between cumulative impoundment and population (see supporting information); their analysis implies a maximum additional water impoundment corresponding to an additional GMSL fall of 6 mm. We plot the future construction suggested by Zarfl et al. (2015) alongside this relationship in Figure S5 and show that the extrapolation to 2040 based on Kopp et al. (2014) underestimates impoundment by 8.4 mm equivalent GMSL fall. There are uncertainties in our estimates based on the Zarfl et al. (2015) database. However, it is clear that the previously derived relationship between cumulative impoundment and world population is likely to significantly underestimate future artificial impoundment contributions to sea level changes.

Finally, we show in detail predictions for two areas that the Zarfl et al. (2015) database indicates will experience significant increases in reservoir impoundment: Southeast Asia and southeastern Brazil (Figure 4). These relatively low‐elevation, highly populated areas near the coast will experience a sea level rise due to impoundment of as much as ~10 mm in the next two decades that will enhance the hazard associated with GMSL rise. Vitousek et al. (2017) suggest that in tropical areas, sea level rise of as little as 50 mm can double the coastal flooding hazard. Thus, major reservoir construction near such coasts could change the frequency of flooding in these populated areas.

## 5 Conclusions

We present an estimate of spatially and temporally resolved sea level change due to the impoundment of water in artificial reservoirs from 1900 to 2011 and a projection of sea level change to the year 2040 due to the same effects. For each year over the period 1900–2011, our predicted global GRD fingerprints are available by download from doi.org/10.5281/zenodo.3751986.

Our analysis of historical data (Lehner et al., 2011a, 2011b) is consistent with previous studies (Chao et al., 2008; Fiedler & Conrad, 2010) that have estimated that globally averaged sea level fell between 21 and 30 mm over the course of the 20th century, representing a significant fraction of the sea level budget. Our spatial analysis is generally consistent with previous work (Fiedler & Conrad, 2010), but the spatio‐temporal resolution of the predictions we present allows for comparison of predictions to records from specific tide gauge sites. Our estimate that reservoir impoundments could have raised sea level by as much as 40 mm should motivate such studies.

Our analysis of reservoirs that are in some form of planning or construction (Zarfl et al., 2015) shows that the era of reservoir construction has not ceased, that this continued impoundment will contribute significantly to changes in sea level, and that the spatial pattern of sea level change will be different from that of the majority of the twentieth century. We show that current best projections may underestimate by nearly a centimeter the effect of water impoundment on GMSL in the next 20 years. It is difficult to accurately predict the sea level fingerprint of reservoirs without precise estimates of the volumetric capacity. Nonetheless, our calculations demonstrate that in areas characterized by a high density of reservoir construction, artificial impoundment is predicted to cause a local rise in sea level, which may change the hazard of coastal flooding. This suggests that an analysis of the local effects on sea level should be performed prior the impoundment of large volumes of water in tropical areas of low elevation, including Southeast Asia and southeastern Brazil.

Finally, our study highlights the importance of establishing a comprehensive database of water impoundment that is volumetrically complete and geospatially and temporally referenced. Artificial water impoundment is a crucial piece of the sea level budget (Cazenave & WCRP Global Sea Level Budget Group, 2018; Kopp et al., 2014, 2015), and accurately accounting for its spatially and temporally varying contribution is imperative. The database would be important not only for the community concerned with the impacts of sea level rise but also to a number of others, including those focused on assessments of global electricity generation (Zarfl et al., 2015), the impact of river fragmentation on ecosystems (Grill et al., 2015), and hazards related to reservoir‐induced seismicity (e.g., Gupta, 2002). In the absence of such a database, our analysis represents the most complete estimate to date of sea level change due to artificial impoundment of water from 1900 to 2040.

## Supporting information

Supporting Information S1

## Data Availability

Satellite altimetry products courtesy of the USDA/NASA G‐REALM program at https://ipad.fas.usda.gov/cropexplorer/global_reservoir/. Data for reservoirs maintained by the U.S. Bureau of Reclamation and accessed via their website at https://www.usbr.gov. GRD Fingerprints for the years 1900–2011 are provided at doi.org/10.5281/zenodo.3751986. The code used for this analysis may be found at https://github.com/wbythewood/ReservoirSeaLevel. GMT was used to create many of the figures in this manuscript (Wessel et al., 2013).
