# Supplementary material for: A Spatially Variable Time Series of Sea Level Change Due to Artificial Water Impoundment
Source: Earths Future. 2020 Jul 25;8(7):e2020EF001497. doi: 10.1029/2020EF001497 (PMC7507774; doi:10.1029/2020EF001497)
Supplement: Supplementary file 1 — Supporting Information S1 [file EFT2-8-e2020EF001497-s001.docx]

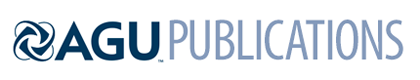


*Earth’s Future*

Supporting Information for

**A Spatially Variable Time Series of Sea Level Change Due to Artificial Water Impoundment**

**William B. Hawley^1,2, †^, Carling C. Hay^3^, Jerry X. Mitrovica^4^, and Robert E. Kopp^5^**

^1^Department of Earth and Planetary Science, University of California, Berkeley, Berkeley, CA 94720, USA.

^2^Berkeley Seismological Laboratory, 215 McCone Hall, University of California, Berkeley, Berkeley, CA 94720, USA.

^3^Department of Earth and Environmental Sciences, Boston College, Chestnut Hill, MA 02467, USA.

^4^Department of Earth and Planetary Sciences, Harvard University, 20 Oxford Street, Cambridge, MA, 02138, USA.

^5^Department of Earth and Planetary Sciences and Rutgers Institute of Earth, Ocean, and Atmospheric Sciences, Rutgers University, New Brunswick, NJ 08901, USA

^†^Now at Lamont-Doherty Earth Observatory of Columbia University, 61 Rte. 9W, Palisades, NY 10964, USA.

Corresponding author: William Hawley (whawley@ldeo.columbia.edu)

**Contents of this file**

Text S1

Figures S1 to S5

Text S1.

The following figures provide additional context for the spatial and temporal variability of changes in sea level due to reservoir construction.

The calculated change in sea level due to the construction of a single reservoir is shown in Fig. S1. The Manicouagan reservoir, in Québec, Canada, is the sixth-largest reservoir in the GRanD database, with a nominal capacity of 141,850 x 10^6^ m^3^, which causes a GMSL fall of ~0.416 mm. The calculated far-field sea level fall for this reservoir is ~0.563 mm, or ~35% larger than the associated GMSL fall.

A comparison between the sea level changes presented in this study and those in Fiedler and Conrad (2010) is shown in Fig. S2. Because their model does not include any GMSL fall, we remove GMSL from our model for this comparison. We predict more sea level rise near where reservoirs are built, likely primarily because we use a database that incorporates ~25% more impounded water. We also show a correspondingly larger sea level fall in areas far from reservoir construction.

Many of the tide gauge locations that experienced large yearly changes did not have a tide gauge in operation at the right time. Stenungsung and Uddevalla, Sweden, began operation in 1962 and 2010, respectively, and did not capture the event in 1934. Instead, we show in Fig. S3a a nearby tide gauge in Smögen, Sweden that was in operation. The other two large predicted sea level change events in Tema, Ghana and Cendering, Malaysia are shown in Fig. S3b and S3c. Though the tide gauges there were in operation during the event, the data do not span enough time to identify a sea level change associated with reservoir construction.

Additionally, we use satellite altimetry from the USDA Global Reservoir and Lake Elevation Database (https://ipad.fas.usda.gov/cropexplorer/global_reservoir/) to estimate fluctuations in two large reservoirs in the GRanD database, shown in Fig. S4. We use these estimates to modify the global fingerprint time series, and plot the resulting sea level curves for the grid point closest to each reservoir, which is not along the coast in either case. We choose this point because it shows the largest signal.

Finally, we show in Fig. S5 a comparison of the predictions presented in this paper with other estimates of GMSL change. We compare the total impounded volume estimated in the GRanD database (Lehner *et al*., 2011) with that of the WRD, used in Chao *et al*. (2008), to show that GRanD records 72% of the impoundment recorded in WRD, but the different tabulations show similar trends. Furthermore, following the approach of Kopp *et al*., (2014) and Rahmstorf *et al*. (2012), we find the relationship between cumulative impoundment and world population assuming a sigmoidal model:

$$V\left( P \right)=a\mathrm{erf} \left( \frac{P-b}{c} \right)+d$$

where *V* is the impoundment, expressed in mm equivalent GMSL fall, *P* is global population, and *a*, *b*, *c*, and *d* are constants. Extrapolating to 2040 (~9.2 billion population), this relationship shows expected impoundment to be 33.3 mm equivalent GMSL fall, an increase of 1.4 mm from the WRD data point in 2010, 31.9 mm equivalent sea level fall. Extending the impoundment data to include the predictions from Zarfl *et al*. (2015) to 2040 shows that if all the reservoirs in that database are built, the GMSL fall by 2040 will reach 41.7 mm, exceeding the model by 8.4 mm. We caution that there are uncertainties inherent in using the Zarfl *et al*. (2015) that arise from (1) the possibility that not all of these reservoirs will be built, and (2) the fact that the database does not include the expected impounded volume, but rather power generation capacity, which we have used to estimate the volume following Grill *et al*. (2015). Nevertheless, our result shows that future impoundment will likely exceed the commonly used estimate from the sigmoidal model fit.

**Figure S1.** Sea level fingerprint of the Manicouagan Reservoir. (**a**) Global map. Maximum predicted sea level fall in the far-field is 0.563 mm, or ~35% larger than the GMSL fall estimate of 0.416 mm. (**b**) Regional map of the sea level fingerprint. Within much of the St. Lawrence seaway, sea level rise is in excess of 10 mm (thin black contour).

**Figure S2.** Comparison between our estimates and Fiedler and Conrad (2010). Both databases in this case are from 1900 to 2009. Positive values indicate where our model estimates more sea level rise than the Fiedler and Conrad (2010) model. Because the total volume of impounded water is different between the datasets, we have removed the GMSL change from our model for this figure.


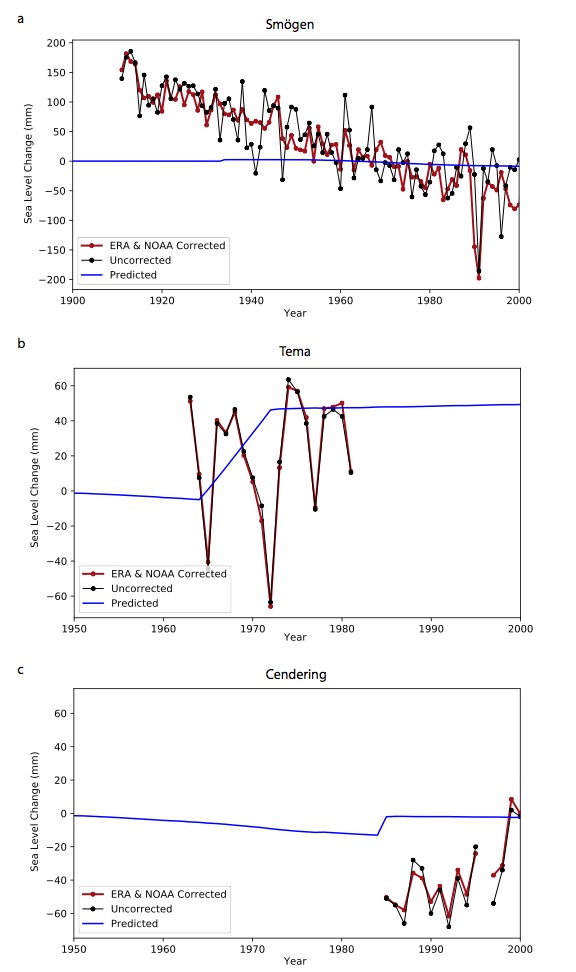


**Figure S3.** Predicted sea level change due to reservoir construction (blue) versus RLR PSMSL observed (black dots) and corrected sea level data (Piecuch *et al*., 2019; red points) at various tide gauges that were in operation during a period of time with significant predicted annual sea level change. (**a**) A tide gauge at Smögen, Sweden, near Stenungsund and Uddevalla, was in operation during the impoundment in 1934, though more distant from the reservoir. (**b**) A tide gauge at Tema, Ghana did not capture much data before or after reservoir construction. (**c**) A tide gauge at Cendering, Malaysia began operation just as the reservoir was completed.


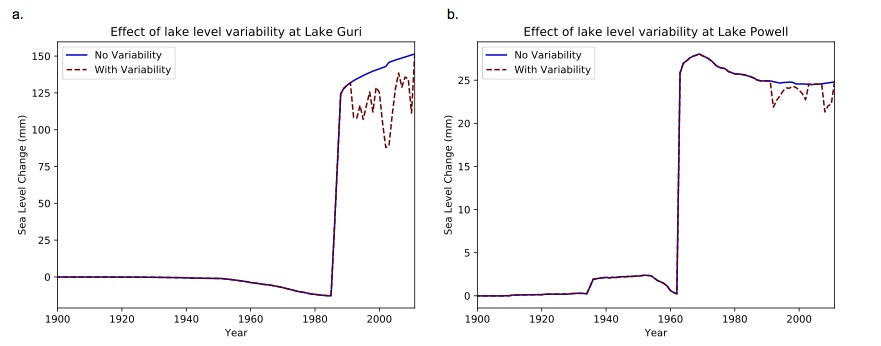


**Figure S4.** The effect of variability of reservoir level on sea level curves at two reservoirs, (**a**) Guri in (7^th^ largest in GRanD), and (**b**) Powell in the United States (43^rd^ largest in GRanD). In both plots, the solid blue line shows the relative sea level change assuming the reservoir remains full; the red dashed line is computed using estimates of variability from satellite altimetry, which begins in the early 1990s. Sea level is shown for the closest grid point to the reservoir location.

**Figure S5.** Estimating future impoundment based on world population. Water impoundment is shown as a function of world population for the GRanD reservoir (Lehner *et al*., 2011; red points), the dataset primarily used in this study, and for Chao *et al*. (2008; black points). Using population projections from the U.N. Department of Economics and social Affairs (2013), and adding the volume of impounded water reported in Zarfl *et al*. (2015) to Chao *et al*. (2008), we have plotted the estimated impoundment vs. population in 2030 and 2040 (purple points). An extrapolation, modeled after Kopp *et al*. (2014; blue dashed line), underestimates the 2040 impoundment by ~8.4 mm equivalent sea level fall.
